# Special Issue “Molecular Mechanisms and Pathophysiology of Sepsis”

**DOI:** 10.3390/ijms26199551

**Published:** 2025-09-30

**Authors:** Vlad Pădureanu

**Affiliations:** Department of Internal Medicine, University of Medicine and Pharmacy of Craiova, 200349 Craiova, Romania; vlad.padureanu@umfcv.ro

Sepsis was characterized for more than 20 years as a microbial infection that results in blood leukocyte alterations, tachycardia, tachypnea, and fever (or hypothermia). With fatality rates dropping to 15–25%, sepsis is widely understood to be a dysregulated systemic inflammatory and immunological response to microbial invasion that results in organ damage. With in-hospital death rates close to 30–50%, septic shock is still characterized as sepsis with hyperlactataemia and concomitant hypotension that needs vaso-pressor therapy. Sepsis is frequently linked to protracted inflammation, immune suppression, organ damage, and lean tissue wasting, rather than an immediate life-threatening condition, thanks to earlier detection and increased adherence to recommended practices. The post-sepsis time is receiving more and more attention, and the post-acute phase of this devastating syndrome is characterized by long-term effects derived from the acute systemic infection. Patients who survive sepsis also suffer long-term cognitive and functional disabilities and a persistent risk of death after discharge. Although in-hospital mortality has decreased due to earlier recognition and better application of optimal practices, immunomodulatory drug use has so far produced unsatisfactory results. In a similar vein, no biomarker can definitively identify sepsis or forecast how it will manifest clinically. Improvements in sepsis outcomes are expected to remain gradual and gradual due to its complexity.

The exceedingly complicated illness known as sepsis is brought on by a host’s reaction to infection, becoming dysregulated. Because of its high mortality and morbidity, sepsis has just been designated a global health priority by the World Health Organization. Sepsis must be identified quickly in order to prevent negative consequences and lower mortality by initiating therapy as soon as possible. In fact, it has been calculated that the death rate from sepsis increases by 7–10% for every hour that treatment is delayed. However, because sepsis lacks distinct signs and symptoms, early diagnosis is still difficult.

At its core, sepsis is an inflammatory illness caused by the innate immune system becoming activated. The innate immune response in sepsis is characterized by two main findings. According to the first study, sepsis is typically brought on by complement and certain cell-surface receptors on cells whose main function is surveillance [1], simultaneously recognizing several infection-derived microbial agents and endogenous danger signals. These cells, which are physically situated where they may continuously sample their local environment, include immunological, epithelial, and endothelial populations. A complex intracellular signaling system with redundant and complementary activities is induced when pathogen-associated molecular patterns (PAMPs) or damage-associated molecular patterns (DAMPs) bind to complement, Toll-like receptors, nucleotide-binding oligomerization domain (NOD)-like receptors, retinoic acid-inducible gene (RIG)-like receptors, mannose-binding lectin, and scavenger receptors, among other receptors [2].

The second important discovery in sepsis is that the expression of several common gene classes involved in inflammation, adaptive immunity, and cellular metabolism is ultimately triggered by the activation of these signaling pathways. To put it another way, the identification of numerous bacteria, viruses, and fungal components, along with host products of tissue damage, results in the recruitment of pro-inflammatory intermediates. These intermediates then phosphorylate mitogen-activated protein kinases (MAPKs), Janus kinases (JAKs), or signal transducers and activators of transcription (STATs), and nuclear translocation of nuclear factor-κB (NF-κΒ), to mention a few.

Numerous early activation genes, including cytokines linked to inflammation (such as tumor necrosis factor (TNF), IL-1, IL-12, IL-18, and type I interferons (IFNs), are expressed when NF-κB is nuclear translocated and its promoter is activated. In addition to polarizing and suppressing adaptive immunity components, these cytokines start a chain reaction of other inflammatory cytokines and chemokines, such as IL-6, IL-8, IFNγ, CC-chemokine ligand 2 (CCL2), CCL3, and CXC-chemokine ligand 10 (CXCL10).

The endothelium changes from an anticoagulant state (in health) to a procoagulant state (in sepsis) due to changes in the expression of several procoagulant and anticoagulant proteins, such as thrombomodulin, tissue factor, von Willebrand factor, plasminogen activator inhibitor 1 (PAI-1), and activated protein C. Vascular endothelial (VE)-cadherin internalization brought on by pro-inflammatory proteases results in the loss of endothelial tight junctions and an increase in vascular permeability [3].

In this Special Issue (SI) of the *International Journal of Molecular Sciences* (IJMS), entitled “Molecular Mechanisms and Pathophysiology of Sepsis”, we highlighted the latest findings regarding immune dysregulation, myocardial dysfunction, adrenal damage, biomarkers, and neonatal fungemia. Five original articles and three critical reviews make up this SI, which is well-balanced. In addition to highlighting knowledge gaps and potential avenues for future research, these papers summarize recent results in the subject of sepsis. The reviews cover important directions such as (i) biomarkers as beacons: sepsis-associated hepato-renal injury [4]; (ii) the role of scavenger receptor BI in sepsis [5]; (iii) neonatal fungemia by non-candida rare opportunistic yeasts [6].

A thorough transcriptome landscape of early-stage sepsis is shown by Taha S. et al., which demonstrates dual-pattern immune dysregulation marked by innate immune hyperactivation and simultaneous inhibition of adaptive immunity, translation, and metabolism [7]. While downregulated programs demonstrated extensive deficits in antigen presentation, protein synthesis, and energy metabolism, upregulated genes and pathways highlighted excessive neutrophil activation, inflammatory signaling, and innate immune priming. Findings that identify actionable gene networks open the door to tailored treatments and point-of-care diagnostic technologies, which could revolutionize the way sepsis is managed in critical care settings.

According to Harvey BI et al., there are sex-based variations in cardiac failure after direct LPS infusion, with male hearts having noticeably lower LV contractile performance than female hearts [8]. Estrogen itself might not have much of an impact on female hearts in response to direct LPS injection, since the proestrus/estrus and metestrus/diestrus groups showed similar amounts of LPS-induced cardiac dysfunction. The mechanisms behind sex-mediated LPS-induced cardiac failure require more investigation, especially the mitochondrial elements causing myocardial variations. New treatments for septic cardiomyopathy may be possible if these significant unknowns are resolved.

In a diverse group of burn patients at high risk of sepsis, Schiavello M et al. [9] propose the possible use of certain circulating EV-miRNAs, including miR-483-5p, -miR-193a-5p, and -miR-188-3p, as biomarkers for the diagnosis of sepsis. Additionally, researchers found that some EV-miRNAs (miR-1-3p, miR-34a-3p, and miR-193a-5p) change depending on how sepsis progresses, while other EV-miRNAs (miR-34a-3p and miR-193a-5p) are linked to the clinical severity of sepsis. These newly discovered circulating indicators may prove to be useful in the future for both prognostic classification and the identification of sepsis in burn victims.

Halimi F et al. used the following sepsis models to compare the transcriptome profiles in the livers of mice and pigs: lipopolysaccharide (LPS)-induced endotoxemia in pigs, which causes sterile inflammation; and cecal ligation and puncture (CLP) in mice and fecal instillation (FI) in pigs, both of which cause polymicrobial septic peritonitis [10]. The pig FI model more nearly reflects the metabolic dysfunction seen in the CLP liver than the pig LPS model, despite the fact that the elevated inflammatory pathways were equally activated in the two pig models. With a typical inflammatory signature among the upregulated genes and metabolic dysfunction among the downregulated genes, the two porcine sepsis models generally resemble the mouse CLP model, according to this thorough comparison of the hepatic responses in mouse CLP versus LPS or FI in pigs. The swine models were unable to reproduce the hepatic ER stress shown in the murine model. The pig FI model is more similar to the mouse CLP model than the pig LPS paradigm when it comes to examining metabolic dysfunction in the liver during sepsis.

Mrozewski L et al. looked into how C5a affected apoptosis and PC12 signaling. In PC12 cells, incubation with 10 nM C5a caused caspase activation via a mechanism that was dependent on C5aR. JNK/MAPK, ERK/MAPK, p38/MAPK, and AKT (PI3K) signaling that is dependent on C5aR was triggered by exposure to sepsis levels of C5a (10 nM). Additionally, after 24 h of exposure to 10 nM C5a, the expression of proteins linked to apoptosis, inflammation, and cell survival increased while that of proteins linked to adhesion, chemotaxis, and anti-inflammatory actions decreased. These findings shed light on the process by which C5a triggers PC12 cell apoptosis and cellular signaling. These results imply that the mechanism of adrenal impairment in sepsis may be significantly influenced by increased complement C5a levels [11].

Early detection and effective treatment are essential for lowering the mortality and morbidity of sepsis, a potentially fatal illness. Numerous putative sepsis biomarkers implicated in various disease processes have been evaluated. Furthermore, new and potential biomarkers such as non-coding RNAs have been developed as a result of recent technological advancements [12]. Given that the only available therapy options for sepsis patients are antibiosis, focus eradication, and critical care support, it is clear that new medicines are required. Characterizing the mechanistic features linked to the onset or course of sepsis would aid in the development of novel therapeutic approaches because the anamneses of sepsis patients are quite diverse. In order to prevent or treat the progression of sepsis, publications that clarify the molecular pathways in vitro or in vivo in preclinical models are invited, as are patient studies that offer insights into novel therapeutic ideas.

This Special Issue, “Molecular Mechanisms and Pathophysiology of Sepsis,” published eight papers, including five original research papers and three review articles, all of which addressed different aspects of sepsis, biomarkers, molecular mechanisms and pathophysiology, and animal models.

We would like to express our full appreciation to the authors who contributed papers to this Special Issue. The remarkable quality of these studies will certainly add new content and generate new openings in multidisciplinary research into molecular mechanisms and pathophysiology of sepsis.

## References

[1] Takeuchi O., Akira S. (2010). Pattern recognition receptors and inflammation. Cell.

[2] Tang D., Kang R., Coyne C.B., Zeh H.J., Lotze M.T. (2012). PAMPs and DAMPs: Signal 0s that spur autophagy and immunity. Immunol. Rev..

[3] Parikh S.M. (2013). Dysregulation of the angiopoietin–Tie-2 axis in sepsis and ARDS. Virulence.

[4] Pasare M.A., Prepeliuc C.S., Grigoriu M.G., Miftode I.L., Miftode E.G. (2025). Biomarkers as Beacons: Illuminating Sepsis-Associated Hepato-Renal Injury. Int. J. Mol. Sci..

[5] Hao D., Xue J.Y., Wang Q., Guo L., Li X.A. (2024). The Role of Scavenger Receptor BI in Sepsis. Int. J. Mol. Sci..

[6] Mpakosi A., Cholevas V., Meletiadis J., Theodoraki M., Sokou R. (2024). Neonatal Fungemia by Non-Candida Rare Opportunistic Yeasts: A Systematic Review of Literature. Int. J. Mol. Sci..

[7] Taha S., Bindayna K., Aljishi M., Sultan A., Almansour N. (2025). Transcriptomic Profiling Reveals Distinct Immune Dysregulation in Early-Stage Sepsis Patients. Int. J. Mol. Sci..

[8] Harvey B.I., Yoniles A.M., Monsivais A., Du J., Zadorozny L., Yu Q., Wang M. (2025). Sex-Specific Differences in LPS-Induced Rapid Myocardial Dysfunction. Int. J. Mol. Sci..

[9] Schiavello M., Bosco O., Vizio B., Sciarrillo A., Pensa A., Pivetta E., Morello F., Risso D., Montrucchio G., Mariano F. (2025). Profiling of miRNAs Contained in Circulating Extracellular Vesicles and Associated with Sepsis Development in Burn Patients: A Proof-of-Concept Study. Int. J. Mol. Sci..

[10] Halimi F., Vanderhaeghen T., Timmermans S., Croubels S., Libert C., Vandewalle J. (2024). Comparative Analysis of Hepatic Gene Expression Profiles in Murine and Porcine Sepsis Models. Int. J. Mol. Sci..

[11] Mrozewski L., Tharmalingam S., Michael P., Kumar A., Tai T.C. (2024). C5a Induces Inflammatory Signaling and Apoptosis in PC12 Cells through C5aR-Dependent Signaling: A Potential Mechanism for Adrenal Damage in Sepsis. Int. J. Mol. Sci..

[12] Agnello L., Ciaccio M. (2023). Biomarkers of Sepsis. Diagnostics.

